# Multi-Objective Ant Colony Optimization Based on the *Physarum*-Inspired Mathematical Model for Bi-Objective Traveling Salesman Problems

**DOI:** 10.1371/journal.pone.0146709

**Published:** 2016-01-11

**Authors:** Zili Zhang, Chao Gao, Yuxiao Lu, Yuxin Liu, Mingxin Liang

**Affiliations:** 1 College of Computer and Information Science & College of Software, Southwest University, Chongqing 400715, China; 2 School of Information Technology, Deaken University, Locked Bag 20000, Geelong, VIC 3220, Australia; 3 Key Laboratory of Symbolic Computation and Knowledge Engineering of Ministry of Education, Jilin University, Changchun 130012, China; Kyushu University, JAPAN

## Abstract

Bi-objective Traveling Salesman Problem (bTSP) is an important field in the operations research, its solutions can be widely applied in the real world. Many researches of Multi-objective Ant Colony Optimization (MOACOs) have been proposed to solve bTSPs. However, most of MOACOs suffer premature convergence. This paper proposes an optimization strategy for MOACOs by optimizing the initialization of pheromone matrix with the prior knowledge of *Physarum*-inspired Mathematical Model (PMM). PMM can find the shortest route between two nodes based on the positive feedback mechanism. The optimized algorithms, named as iPM-MOACOs, can enhance the pheromone in the short paths and promote the search ability of ants. A series of experiments are conducted and experimental results show that the proposed strategy can achieve a better compromise solution than the original MOACOs for solving bTSPs.

## Introduction

Multi-objective traveling salesman problem (MOTSP), as one of the typical multi-objective optimization problems (MOOP), is an important field in operations research and networks [1]. Networks form the backbone of many complex systems, ranging from the Internet to human societies [2]. And network models have been widely employed [3]. Lots of real-world problems, such as multi-objective network structure design problems and multi-objective vehicle routing problems, can be formulated as MOTSPs [4, 5]. Establishing an efficient approach to find a set of solutions with good trade-off among different objectives for a MOTSP has great practical significance. As a colony-based optimization approach, Multi-Objective Ant Colony Optimization (MOACOs) can obtain a certain number of trade-off solutions in a single run. And MOACOs are suitable and have been widely applied for solving multi-objective optimization problems [6–8]. In the past two decades, many researches of MOACOs have been presented. For example, BicriterionAnt algorithm (BIANT) has been proposed to solve bi-criteria vehicle routing problems [9], Pareto Ant Colony Optimization (PACO) has been designed to solve the multi-objective portfolio selection problems [10], and Multiple Ant Colony System (MACS) has been proposed to solve the vehicle routing problems with time windows [11]. García-Martínez et al. [12] have discussed a taxonomy of MOACOs according to the number of heuristic matrices and pheromone matrices. According to the research of García-Martínez et al. [12], MOACOs have been used to carry out MOTSPs and some guidelines on how to design MOACOs are proposed. However, due to the disturbance of non-global optimal paths, MOACOs often cannot achieve a good trade-off solution or fall into the local optimal solutions [13].

Currently, a unicellular and multi-headed slime mold, *Physarum polycephalum*, shows an ability to form self-adaptive and high efficient networks in biological experiments [14–16]. Tero et al. [17] have captured the positive feedback mechanism of *Physarum* in foraging and have built a *Physarum*-inspired mathematical model (PMM). The edges of *Physarum* network are seemed as tubes with flux flowing in PMM. Tubes with a large flux will grow, while those with a small flux will disappear. Based on this dynamic behavior of tube diameter, PMM exhibits a unique feature of critical paths reserved in the process of network evolution. If prior knowledge exists or can be generated at a low computational cost, good initial estimates may generate better solutions with faster convergence [18]. Taking advantage of the prior knowledge of PMM, Zhang et al. [19] have proposed an optimization strategy for updating the pheromone matrix of ACO with one or multiple objectives. However, the optimization of pheromone matrix in each step will cause much computational cost. Meanwhile, a bi-objective TSP, shorted as bTSP, is simplified to a single objective one when Zhang et al. measure the performance of their strategy. What’s more, using an unchanged prior knowledge to update pheromone matrix which changes with the deepening of search, solutions could suffer premature convergence. Therefore, in this paper, we propose a new strategy with the prior knowledge of PMM. In order to improve computational efficiency, the new strategy updates pheromone matrix in the initialization of MOACOs. Furthermore, we estimate the optimization strategy for three MOACOs, i.e., PACO [10], MACS [11] and BIANT [9], and validate the performance of these three algorithms in four bi-objective symmetric TSP instances using five typical MOTSP measurements.

The paper is organized as follows. The section of Problem statement introduces some definitions about MOOP and bTSP. Specially, we define five typical measurements to estimate the performance of algorithms when solving bTSPs. The section of *Physarum*-inspired mathematical model presents the basic ideas of original PMM with one pair of inlet/outlet nodes, then proposes an improved PMM with multi-pair of inlet/outlet nodes. The section of PMM-based MOACOs first introduces the principles of three typical MOACOs for solving bTSPs, and then presents the formation of optimized algorithms based on PMM. The section of Results estimates and compares the computational efficiency of optimized MOACOs and the traditional MOACOs for solving bTSPs. The section of Conclusions concludes this paper.

## Problem statement

This section first introduces the basic concepts of MOOP, then gives the definition and measurements of bTSPs.

### (1) Basic concepts of MOOP

A MOOP deals with two or more objective functions simultaneously. As usual, a MOOP can be mathematically formulated as

$$\begin{array}{c} \left(MOOP\right)=\{\begin{array}{c} \min F\left(x\right)=(f_{1}\left(x\right),f_{2}\left(x\right),...,f_{K}\left(x\right))\\ s.t.x\in D \end{array} \end{array}$$ (1)

where *D* is the feasible solution space, *F*(*x*) is consisted of *K* objective functions *f*_*k*_, *k* = 1, …, *K*.

Since different objectives in a MOOP are usually conflicting, it is impossible to find one best solution that can optimize all objectives simultaneously [12]. Instead, there may exists a number of solutions in the solution space in which no solution is superior to others for all objectives. The goal of a MOOP is to obtain these non-dominated solutions with good trade-offs among different objectives, which are named as Pareto set. The related definitions [20] are as follows.

**Definition 1** Suppose *x*_1_, *x*_2_ ∈ *D*, then *x*_1_ is said to be dominated by *x*_2_, denoted as *x*_2_ ≺ *x*_1_, if and only if ∀*i* ∈ 1, …, *K*, *f*_*i*_(*x*_2_) ≤ *f*_*i*_(*x*_1_) and ∃*i* ∈ {1, …, *K*}, *f*_*i*_(*x*_2_) < *f*_*i*_(*x*_1_).

**Definition 2** If a solution is not dominated by any other solutions in *D*, then it is named as a Pareto optimal solution or non-dominated solution. The set of all the Pareto optimal solutions is named as the Pareto set (PS), i.e., PS = {*x* ∈ *D*|∄*y* ∈ *D*, *F*(*y*) ≺ *F*(*x*)}.

**Definition 3** The image of the PS in the objective space is named as the Pareto front (PF), i.e., PF = {*F*(*x*)|*x* ∈ PS}.

For a MOOP instances, the true PS is always not known [21]. Instead, the pseudo-optimal PS is defined as an approximation of the true PS, which is obtained by fusing all PSs returned by all existing algorithms in several runnings [22].

### (2) Definition and measurements of a bTSP

As an extension of a single objective TSP, bTSP manages two objectives simultaneously, which can be described as follows. Let *G* = (*V*, *E*) be a complete weighted graph where *V* = {1, …, *n*} is a set of *n* cities, and *E* = {(*i*, *j*)|*i*, *j* ∈ *N*, *i* ≠ *j*} is a set of edges fully connecting cities *V*. Each edge is assigned two different values. $w_{ij}^{k}$ represents the value factor between nodes *i* and *j* for objective *k*, where *k* ∈ {1, 2}. The solution of a bTSP is to obtain a set of non-dominated Hamiltonian tours (denoted as Ω) that approximates the pseudo-optimal PS. In a bTSP, the objective function *f*_*k*_ can be defined as:

$$\begin{array}{c} f_{k}\left(x\right)=w_{x_{n}x_{1}}^{k}+\sum_{i=1}^{n-1}w_{x_{i}x_{i+1}}^{k},\;x\in \Omega \end{array}$$ (2)

where *x*_*i*_ represents the *i*^*th*^ city in the Hamiltonian tour *x*, and *x*_*i*_∈*V* [23].

Five typical measurements based on the definition of García-Martínez [12] are used to estimate the performance of bTSP solution algorithms:

1. The graphical representation of PF returned by an algorithm. These graphics provide a visual information for estimating the quality and distribution of solutions. It is an intuitive measurement of PF with a graphical representation, if there are two PFs, *PF*_*A*_ and *PF*_*B*_, and the results of *PF*_*A*_ converge to the bottom-left region comparing with those of *PF*_*B*_, we can deduce that the results of *PF*_*A*_ are better than those of *PF*_*B*_.
2. M1 metric represents the distance between the result of an algorithm, denoted as *Y*, and the pseudo-optimal Pareto front ($\bar{Y}$). This matric is based on Eq (3), in which |*Y*| means the number of non-dominated solutions in front of *Y*. The smaller *M*_1_ metric is, the smaller difference between $\bar{Y}$ and *Y* is.
  $$\begin{array}{c} M_{1}\left(Y\right)=\frac{1}{\left|Y\right|}\sum_{p\in Y}\min\{∥p-\bar{p}∥;\bar{p}\in \bar{Y}\} \end{array}$$ (3)
3. *M*_2_ metric evaluates the distribution of solutions in the PF returned by an algorithm (denoted as *Y*). This metric is based on Eq (4), in which parameter *σ* is a positive constant. The larger *M*_2_ metric is, the wider the coverage of the obtained solutions is.
  $$\begin{array}{c} M_{2}\left(Y\right)=\frac{1}{\left|Y-1\right|}\sum_{p\in Y}\left|\left\{q\in Y;∥p-q∥\;>\sigma \right\}\right| \end{array}$$ (4)
4. *M*_3_ metric is used to to evaluate the diameter of PF returned by an algorithm (denoted as *Y*) based on Eq (5), in which *p*_*i*_ denotes the solution value in *p* for objective *i*. The larger *M*_3_ metric is, the larger region of the objective space of solutions locate.
  $$\begin{array}{c} M_{3}\left(Y\right)=\sqrt{\sum_{i=1}^{2}\max\{∥p_{i}-q_{i}∥;p,q\in Y\}} \end{array}$$ (5)
5. *C* metric is devoted to compare the performance of two algorithms by calculating the dominance degree of their respective PF. In Eq (6), *Y*_1_ and *Y*_2_ represent PFs returned by two different algorithms.
  $$\begin{array}{c} C\left(Y_{1},Y_{2}\right)=\frac{|\left\{p_{2}\in Y_{2};\exists p_{1}\in Y_{1}:p_{1}≺p_{2}\right\}|}{|Y_{2}|} \end{array}$$ (6)

## *Physarum*-inspired mathematical model

We first present the basic ideas of original PMM, i.e., PMM with a single pair of inlet/outlet nodes. Then PMM with multi-pair of inlet/outlet nodes is proposed for finding the shortest route that connects multiple food sources.

### (1) The original PMM

The original PMM is used for finding the shortest route between two food sources in a maze [14] or road map [17]. The main idea of PMM contains two empirical rules. First, tubes disappear with a small flux. Second, when more than one tube connects the same nodes, the shorter tubes incline to reserve. Based on these phenomenological rules, the original PMM is established, which can be described as follows.

**Fig 1:**
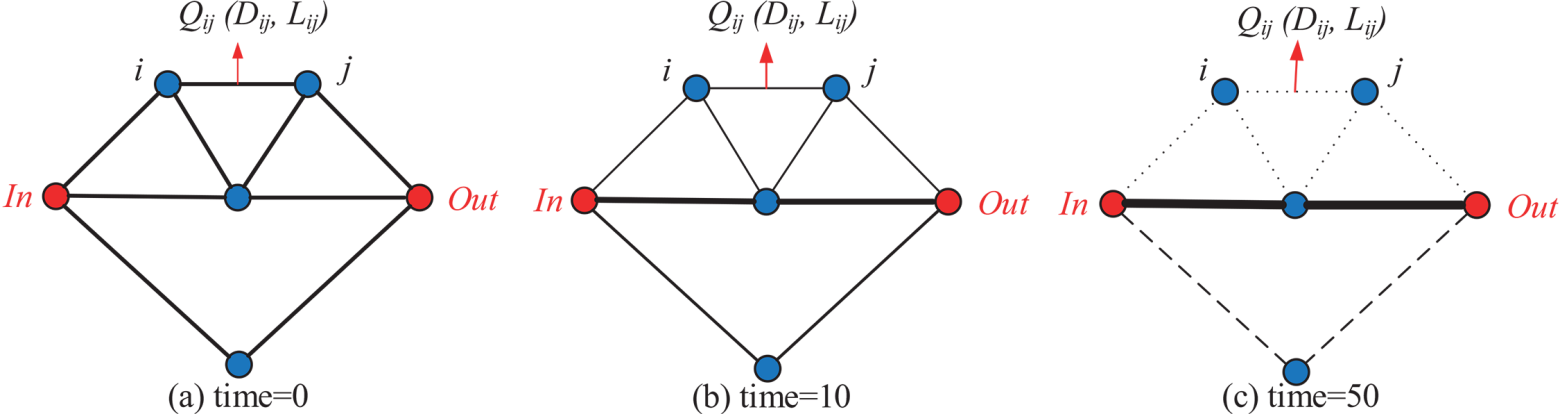
A network example for the presentation of original PMM. (a) The initial network, (b) The intermediate network evolved by the original PMM, and (c) The final network evolved by the original PMM.

Taking Fig 1(a) as an example, each edge in a network represents a tube. A finite quantity of flux *I*_0_ flows from *In* to *Out* through different paths. *In* and *Out* represent the inlet and outlet node of the network, respectively. The variable *Q*_*ij*_ is used to express the flux in the tube (*i*, *j*). Assuming that the flow in the tube approximates to the Poiseuille flow, then the flux *Q*_*ij*_ can be formulated as

$$\begin{array}{c} Q_{ij}=\frac{D_{ij}}{L_{ij}}\left(p_{i}-p_{j}\right) \end{array}$$ (7)

where *L*_*ij*_ represents the length of a tube (*i*, *j*), *p*_*i*_ is the pressure at the node *i*, *D*_*ij*_ is defined as a measure of conductivity, which is correlated with the tube’s thickness.

According to the Kirchhoff Law, the flux input is equivalent to the flux output. Especially, *In* only has output flow and *Out* only has input flow. Hence, the following equation can be obtained.

$$\begin{array}{c} \sum_{i}Q_{ij}=\sum_{i}\frac{D_{ij}}{L_{ij}}\left(p_{i}-p_{j}\right)=\{\begin{array}{cc} -I_{0} & \text{for}\;j=1,\\ I_{0} & \text{for}\;j=2,\\ 0 & \text{otherwise} \end{array} \end{array}$$ (8)

By setting *p*_2_ = 0 as the basic pressure level, all *p*_*i*_ can be calculated by Eq (8). Then, the flux *Q*_*ij*_ is obtained based on Eq (7). For describing the adaptation of tubular thickness with flux, we suppose that the conductivity *D*_*ij*_ changes over time according to the flux *Q*_*ij*_, as shown in Eq (9).

$$\begin{array}{c} \frac{dD_{ij}}{dt}=f(|Q_{ij}\left|\right)-rD_{ij} \end{array}$$ (9)

where *f*(*Q*) is an increasing function with *f*(0) = 0, *r* is the decay rate of tubes. This equation indicates that conductivity is enhanced by the flux increases, and tends to decline if the flux decreases. In this paper, the functional form *f*(*Q*) = |*Q*_*ij*_|/(1 + |*Q*_*ij*_|) and *r* = 1 are adopted. Hence, the adaption Eq (9) is simply expressed by Eq (10) as follow.

$$\begin{array}{c} \frac{D_{ij}^{n+1}-D_{ij}^{n}}{\delta t}=\frac{|Q_{ij}|}{1+|Q_{ij}|}-D_{ij}^{n+1} \end{array}$$ (10)

The new value of *D*_*ij*_ will be fed back to Eq (8). The iteration does not terminate until the constraint $|D_{ij}^{n+1}-D_{ij}^{n}|\leq 10^{-6}$ is satisfied. Fig 1(b) displays the intermediate network and Fig 1(c) shows the final network. It is clear that the core mechanism of PMM is the positive feedback, i.e., greater conductivity leads to greater flux, and this in turn increases conductivity [17]. The tubes that in the shorter paths have a higher flux, they tend to become wider and be reserved in the process of network evolution. While, some longer tubes will become narrower and disappear. Finally, the reserved tubes, denoted as critical paths, will be the solution to a path-finding problem.

### (2) PMM with multi-pairs of inlet/outlet nodes

In order to apply the original PMM for solving a TSP, PMM with multi-pairs of inlet/outlet nodes is proposed in this section. In a cycle, each pair of two food sources is selected as inlet/outlet nodes once. The total flux is set as *F*/*M*, where *M* represents the number of tubes in a network. The length of a tube *L*_*ij*_ is calculated as $L_{ij}=\sum_{k=1}^{K}w_{ij}^{k}/K$. Based on Eqs (7) and (8), the flux $Q_{ij}^{\left(m\right)}$ at the *m*^*th*^ selection can be calculated. Then, the final flux *Q*_*ij*_ of a tube is substituted with the average flux $\bar{Q_{ij}}$, as shown in Eq (11). According to $\bar{Q_{ij}}$, we can update the conductivity of each tube based on Eq (10). The above steps are repeated until the change of conductivity of each tube is less than 10^−6^ [19].

$$\begin{array}{c} \bar{Q_{ij}}=\frac{1}{M}\sum_{m=1}^{M}\left|Q_{ij}^{\left(m\right)}\right| \end{array}$$ (11)

**Fig 2:**
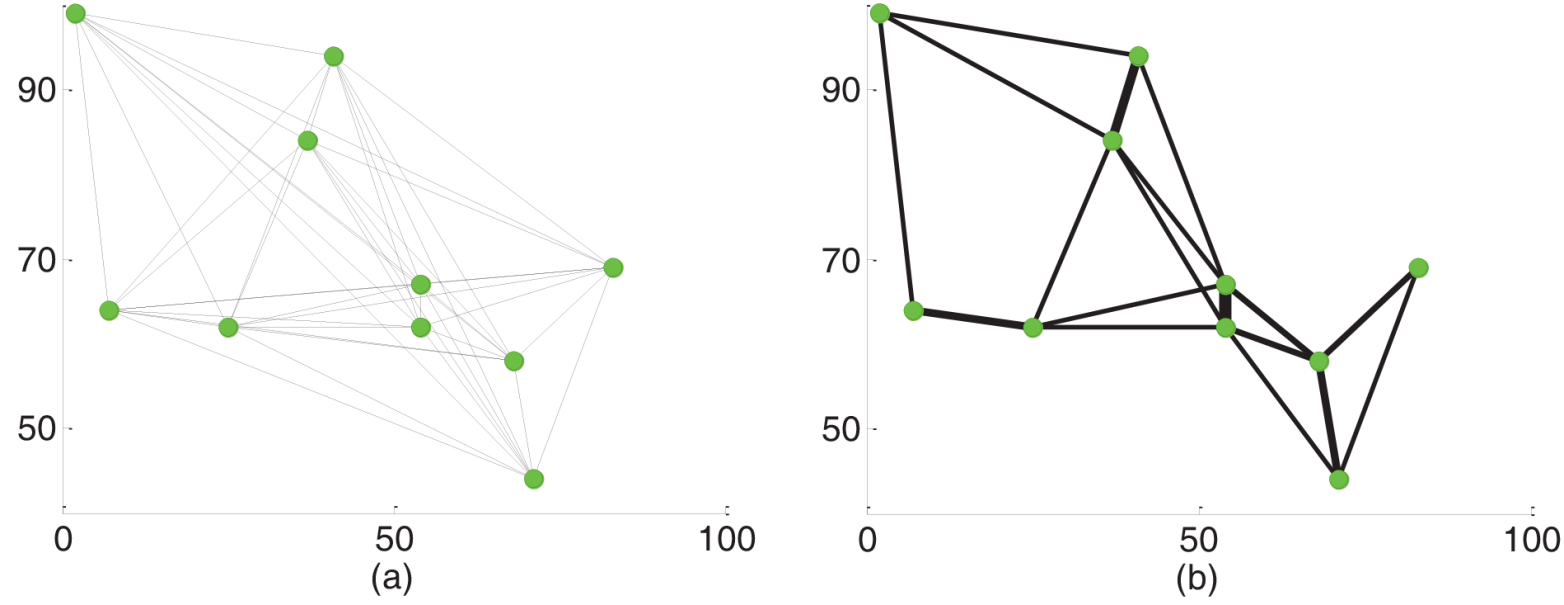
A network example for illustrating the proposed PMM with multi-pair of inlet/outlet nodes. (a) The initial network and (b) The final network evolved by the proposed PMM.

For example, Fig 2(a) is a complete network with ten nodes, and Fig 2(b) is the final network evolved by PMM with multi-pair of inlet/outlet nodes. We find that some shorter tubes will become wider than the initial state and will be reserved ultimately, while other longer tubes will disappear. These reserved tubes are also named as the critical tubes. Taking advantage of critical tubes reserved in the evolution process, the improved PMM is proposed to optimize MOACOs for solving bTSPs in the next section.

## The PMM-based MOACOs

This section first presents three basic principles of MOACOs for solving a bTSP, i.e., ant movement rule, the matrix updating rule for local and global pheromone. Then, we formulate our proposed optimized MOACOs method based on PMM, denoted as iPM-MOACOs.

### (1) MOACOs for solving a bTSP

In the MOACOs-based bTSP, each ant is first randomly put on a city, then it chooses the next unvisited city according to the amount of pheromone in the path based on the **Ant Movement Rule**. Every time an ant travels a city, the amount of pheromone on this path will be updated by this ant. This process is called as the **Local Pheromone Matrix Updating Rule**. Finally, once all ants have finished constructing their routes, the **Global Pheromone Matrix Updating Rule** is implemented. In the following, we will take three typical MOACOs (i.e., PACO [10], MACS [11] and BIANT [9]) as examples to describe three key procedures for solving a bTSP.

#### • Ant Movement Rule

For BIANT, each objective is recorded by a pheromone trail matrix and a heuristic matrix. The ant movement probability $P_{ij}^{h}$ is:

$$\begin{array}{c} P_{ij}^{h}=\{\begin{array}{cc} \frac{{\left[\tau_{ij}^{0}\right]}^{\gamma \alpha }\times {\left[\tau_{ij}^{1}\right]}^{(1-\gamma )\alpha }\times {\left[\eta_{ij}^{0}\right]}^{\gamma \beta }\times {\left[\eta_{ij}^{1}\right]}^{(1-\gamma )\beta }}{\sum_{u\in N_{i}^{h}}{\left[\tau_{iu}^{0}\right]}^{\gamma \alpha }\times {\left[\tau_{iu}^{1}\right]}^{(1-\gamma )\alpha }\times {\left[\eta_{iu}^{0}\right]}^{\gamma \beta }\times {\left[\eta_{iu}^{1}\right]}^{(1-\gamma )\beta }}, & \text{if}\;j\in N_{i}^{h}\\ 0, & \text{otherwise} \end{array} \end{array}$$ (12)

where *γ* = (*h* − 1)/(*s* − 1), *h* is the serial number of an ant and *s* is the total number of ants.

Although PACO and MACS are based on ant colony system (ACS), there are a bit different from ACS. In PACO, two pheromone matrices are considered, and each represents an objective independently. In MACS, it has a signal pheromone matrix and two heuristic matrices. First, *q*_0_ is a predefined parameter (*q*_0_ ∈ [0, 1]), and *q* is a random number uniformly distributed in [0, 1]. Then, an ant *h* located at a city *i* moves to the next city *j* according to the probability $P_{ij}^{h}$, as shown in Eqs (13) and (14).

If *q* ≤ *q*_0_:

$$\begin{array}{c} P_{ij}^{h}=\{\begin{array}{cc} 1,if\;j=argmax_{j\in N_{i}^{h}}\left({\left[\sum_{k=1}^{2}p_{k}\times \tau_{ij}^{k}\right]}^{\alpha }\times \eta_{ij}^{\beta }\right), & \text{for}\;\text{PACO}\\ 1,if\;j=argmax_{j\in N_{i}^{h}}\left(\tau_{ij}^{\alpha }\times {\left[\eta_{ij}^{0}\right]}^{\gamma \beta }\times {\left[\eta_{ij}^{1}\right]}^{(1-\gamma )\beta }\right), & \text{for}\;\text{MACS}\\ 0, & \text{otherwise} \end{array} \end{array}$$ (13)

else:

$$\begin{array}{c} P_{ij}^{h}=\{\begin{array}{cc} \frac{{\left[\sum_{k=1}^{2}p_{k}\times \tau_{ij}^{k}\right]}^{\alpha }\times \eta_{ij}^{\beta }}{\sum_{u\in N_{i}^{h}}{\left[\sum_{k=1}^{2}p_{k}\times \tau_{iu}^{k}\right]}^{\alpha }\times \eta_{iu}^{\beta }}, & \text{if}\;j\in N_{i}^{h},\;\text{for}\;\text{PACO}\\ \frac{\tau_{ij}^{\alpha }\times {\left[\eta_{ij}^{0}\right]}^{\gamma \beta }\times {\left[\eta_{ij}^{1}\right]}^{(1-\gamma )\beta }}{\sum_{u\in N_{i}^{h}}\tau_{iu}^{\alpha }\times {\left[\eta_{iu}^{0}\right]}^{\gamma \beta }\times {\left[\eta_{iu}^{1}\right]}^{(1-\gamma )\beta }}, & \text{if}\;j\in N_{i}^{h},\;\text{for}\;\text{MACS}\\ 0, & \text{otherwise} \end{array} \end{array}$$ (14)

where *α* and *β* weight the importance of pheromone matrix *τ* and the heuristic information *η*, respectively. $N_{i}^{h}$ is a feasible neighbor of the ant *h* in a city *i*. In PACO, the pheromone matrix $\tau_{ij}^{k}$ represents the amount of pheromone in the path connecting cities *i* and *j* for objective *k*. The heuristic information $\eta_{ij}=2/\sum_{k=1}^{2}w_{ij}^{k}$ represents the expectation that ant *h* moves from city *i* to city *j*. *p*_*k*_ weights the importance of objective *k*’s pheromone matrix. In MACS, $\eta_{ij}^{k}$ represents the heuristic information for objective *k*. For each ant *h*, *γ* is computed by *h*/*s*.

#### • Local Pheromone Matrix Updating Rule

For BIANT, there is only one global rule for updating the pheromone matrix, and no local pheromone matrix updating strategy.

For PACO, each pheromone matrix $\tau_{ij}^{k}$ for the objective *k* is updated as follow:

$$\begin{array}{c} \tau_{ij}^{k}=\left(1-\rho \right)\times \tau_{ij}^{k}+\rho \times \tau_{0} \end{array}$$ (15)

where *ρ* is the pheromone evaporation rate, and *τ*_0_ is a constant which represents the initial amount of pheromone.

For MACS, it has a single pheromone matrix *τ*_*ij*_ that is updated as follow:

$$\begin{array}{c} \tau_{ij}=\left(1-\rho \right)\times \tau_{ij}+\rho \times \tau_{0} \end{array}$$ (16)

The value of *τ*_0_ in MACS is determined by the obtained PS, which is initialized by a set of heuristic solutions and calculated by taking their average cost in each of two objective functions *f*_0_ and *f*_1_ based on Eq (17):

$$\begin{array}{c} \tau_{0}=\frac{1}{{\hat{f}}_{0}\times {\hat{f}}_{1}} \end{array}$$ (17)

The value of *τ*_0_ is dynamic change with the evolution of system. Every time an ant *h* builds a complete solution, it is compared to the existing PS to check whether or not the existing PS is a non-dominated solution. When all ants have built a route, $\tau_{0}^{\prime }$ is calculated based on Eq (17) with the average value of each objective function taken from solutions included in the current PS. Then, if $\tau_{0}^{\prime }>\tau_{0}$ (*τ*_0_ means the current initial pheromone value), *τ*_0_ is replaced by $\tau_{0}^{\prime }$. Otherwise, *τ*_0_ is not changed.

#### • Global Pheromone Matrix Updating Rule

For MACS, if $\tau_{0}^{\prime }\leq \tau_{0}$, the global update rule is performed with each solution *S* of the current PS by applying the following rule on its composing paths (*i*, *j*):

$$\begin{array}{c} \tau_{ij}=\left(1-\rho \right)\times \tau_{ij}+\rho \times \Delta \tau_{ij},\;\text{if}\;\left(i,j\right)\in S \end{array}$$ (18)

where

$$\begin{array}{c} \Delta \tau_{ij}=\frac{1}{f_{0}\left(S\right)\times f_{1}\left(S\right)},\;\text{if}\;\left(i,j\right)\in S \end{array}$$ (19)

For BIANT, $\tau_{ij}^{k}$ is first updated by Eq (20) for each path (*i*, *j*).

$$\begin{array}{c} \tau_{ij}^{k}=\left(1-\rho \right)\times \tau_{ij}^{k} \end{array}$$ (20)

Then, each ant that generates a solution in the PS at the current iteration is allowed to update the global pheromone matrices, i.e.,

$$\begin{array}{c} \tau_{ij}^{k}=\tau_{ij}^{k}+\Delta \tau_{ij}^{k} \end{array}$$ (21)

where

$$\begin{array}{c} \Delta \tau_{ij}^{k}=\frac{1}{l} \end{array}$$ (22)

where *l* represents the number of ants taking part in updating the pheromone matrices.

For PACO, the global pheromone matrix updating rule is:

$$\begin{array}{c} \tau_{ij}^{k}=\left(1-\rho \right)\times \tau_{ij}^{k}+\rho \times \Delta \tau_{ij}^{k} \end{array}$$ (23)

where the definition of $\Delta \tau_{ij}^{k}$ can be seen in Eq (24). *f*_*k*_(*best*) and *f*_*k*_(*second*-*best*) denote the minimum total cost of route and the second minimum total cost of route that ants have travelled for objective *k*, respectively.

$$\begin{array}{c} \Delta \tau_{ij}^{k}=\{\begin{array}{cc} \frac{1}{f_{k}\left(best\right)}+\frac{1}{f_{k}\left(second-best\right)}, & \text{if}\;\text{path}(i,j)\in \text{best}\;\text{and}\;\text{second-best}\;\text{solutions}\\ \frac{1}{f_{k}\left(best\right)}, & \text{if}\;\text{path}(i,j)\in \text{best}\;\text{solution}\\ \frac{1}{f_{k}\left(second-best\right)}, & \text{if}\;\text{path}(i,j)\in \text{second-best}\;\text{solution}\\ 0, & \text{otherwise} \end{array} \end{array}$$ (24)

Three procedures compose a life cycle of ant colony. After each cycle, several non-dominated Hamiltonian tours will be generated. As time elapses, some new tours will be established, and may dominate the afore-generated tours. At the end of an algorithm, PS is composed of all non-dominated Hamiltonian tours. The image of this PS is the PF returned by the algorithm for a bTSP. However, PFs returned by most of MOACOs always concentrate on the local optimal regions [13]. Hence, we propose a framework based on PMM to improve the performance of MOACOs.

### (2) The improved MOACOs based on PMM

**Fig 3:**
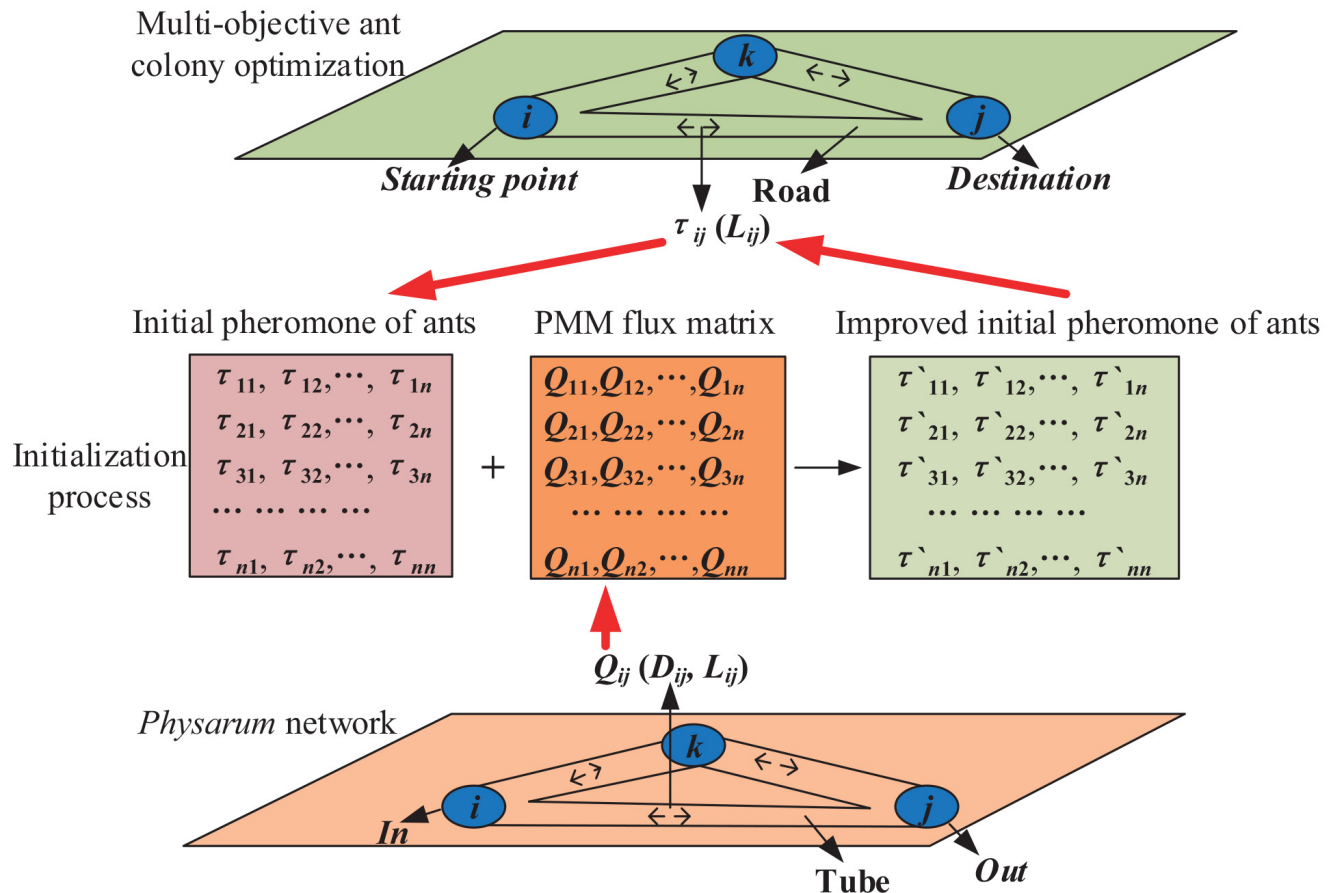
The illustration of working mechanism of iPM-MOACOs. The food sources and tubes of *Physarum* network represent cities and paths in a road network, respectively.

Taking advantage of PMM in path-finding, we propose a series of optimized MOACOs for solving a bTSP, denoted as iPM-MOACOs. In the iPM-MOACOs-based bTSP, we suppose that there is a *Physarum* network with pheromone flows in tubes, as shown in Fig 3. The food sources and tubes of *Physarum* network are defined as cities and paths connecting two different cities, respectively. While differing from the method in [19], which makes use of the prior knowledge of PMM working in the search process, we exploit the prior knowledge of *Physarum* network pheromone matrix to initialize the pheromone matrices of ants. The optimized strategy can improve the search ability of algorithms. There are two advantages in this strategy. First, if the *Physarum* network pheromone matrices are consistent with the optimal solutions, results of optimized strategy are closer to the optimal solutions than those of MOACOs. Second, if the *Physarum* network pheromone matrices are divergent with the optimal solutions, they will expand the search scope of ants. With the number of iteration increasing, more and more ants will select more reasonable paths. And the influences of prior knowledge will decrease with pheromone evaporating.

Compared with the original MOACOs, rules of the ant movement and pheromone matrices updating remain the same. The only difference between the optimized and the original is the initialization of pheromone matrices. MOACOs usually sets the value of pheromone matrices as a fixed value (like 0 or 1) or random digits. When initializing the optimized strategy, the pheromone matrices will be preset with the priori knowledge of PMM. The initialization of iPM-MOACOs are shown in Eqs (25) and (26).

$$\begin{array}{c} \tau_{ij}^{k}=\tau_{ij}^{k}+\frac{\varepsilon \times \bar{Q_{ij}^{k}}}{I_{0}},\;\text{for}\;\text{optimized}\;\text{PACO}\;\text{and}\;\text{BIANT} \end{array}$$ (25)

$$\begin{array}{c} \tau_{ij}=\tau_{ij}+\sum_{k=1}^{2}\frac{\varepsilon \times \bar{Q_{ij}^{k}}}{I_{0}},\;\text{for}\;\text{optimized}\;\text{MACS} \end{array}$$ (26)

where *k* stands for the *k*^*th*^ objective, and *ε* is defined as an impact factor to measure the effect of flowing pheromone in the *Physarum* network, as shown in Eq (27). *Psteps* stands for the total number of iterations affected by PMM, and *λ* ∈ (1,1.2).

$$\begin{array}{c} \varepsilon =1-\frac{1}{1+\lambda^{\frac{Psteps}{2}}} \end{array}$$ (27)

**Fig 4:**
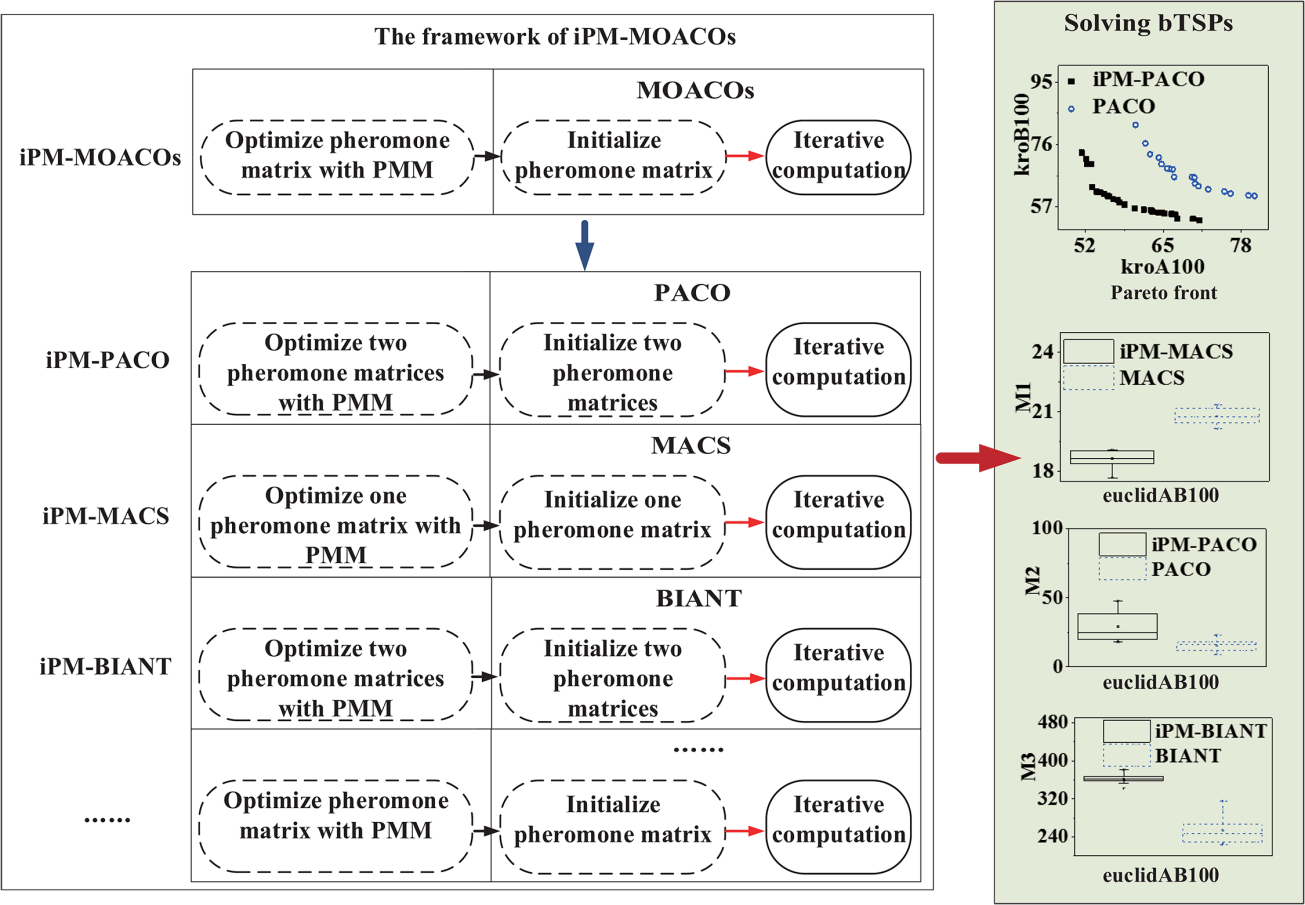
The framework of iPM-MOACOs, which implies our proposed strategies based on the PMM can optimize the initialization of MOACOs.

Fig 4 presents the framework of MOACOs based on PMM. For convenience sake, the optimized algorithm is named as the original algorithm with a prefix ‘iPM-’, for example, iPM-PACO, iPM-MACS and iPM-BIANT.

The pseudocode of iPM-MOACOs for solving a bTSP can be briefed in Table 1, where *Tsteps* represents the total steps of iterations.

**Table 1:** The algorithm of iPM-MOACOs for solving a bTSP.

**Input**:
Graph *G* = (*N*, *E*) with two different weights for each edge
**Output**:
PF
**Begin**
**(i) Initialization**
(a) Initialize the values of parameters and variables
(b) Set the iteration counter *N*: = 0
(c) Initialize the values of pheromone matrices with PMM
**(ii) While** (*N* < *T steps*) **do**
(a) Ant movement
(b) Record the list of cities that ants have traveled and find the PS and PF
(c) Update the pheromone matrix
(d) *N*: = *N*+1
**End while**
**(iii) Output PF**
**End Begin**

## Results

This section first presents instances and parameters used in the experiments. Then, we estimate the performances of MOACOs, PM-MOACOs [19] and iPM-MOACOs for solving bTSPs by five measurements. Furthermore, we discuss the performances of MOACOs and iPM-MOACOs with the hypervolume indicator.

### (1) bTSP instances and parameters

In this section, according to García-Martínez et al. [12], the bi-objective symmetric TSP instances obtain from Jaskiewicz’s web page (https://eden.dei.uc.pt/~paquete/tsp/). Each of these instances is constructed from two different single objective TSP instances with the same number of nodes. More information is provided in [24]. In this paper, we will use four bi-objective TSP instances, i.e., euclidAB100, kroAB100, kroAB150 and kroAB200, to estimate our proposed method.

The parameters are set to the generic values when we apply MOACOs to a bTSP, as shown in Table 2. Especially, the parameter settings among the MOACOs, PM-MOACOs and iPM-MOACOs are the same. All experiments are implemented on PC with 3.2 GHz CPU, 4 GB RAM and Windows 7 OS. In order to wipe off the computational fluctuation, all results in our experiments are averaged over 10 times [12].

**Table 2:** Major parameters and their default values used in this paper.

Symbol	Explanation	Value
*α*	The relative importance of pheromone trail	2 for PACO and BIANT
		4 for MACS
*β*	The relative importance of heuristic information	11 for PACO and BIANT
		8 for MACS
*ρ*	The pheromone evaporation rate	0.1 for PACO
		0.3 for BIANT
		0.2 for MACS
*F*	The amount of pheromone released by each ant	100
*s*	The total number of ants	100
*λ*	A parameter determined the value of *ε*	1.05
*τ*_0_	The initial amount of pheromone in each path	0
*q*_0_	A predefined parameter between 0 and 1	0.1
*D*_*ij*_	The initial value of the conductivity of each tube	1
*Psteps*	The total steps of iteration affected by PMM	300
*Tsteps*	The total steps of iteration	300

### (2) Experimental results

#### • Comparisons between MOACOs and iPM-MOACOs

**Fig 5:**
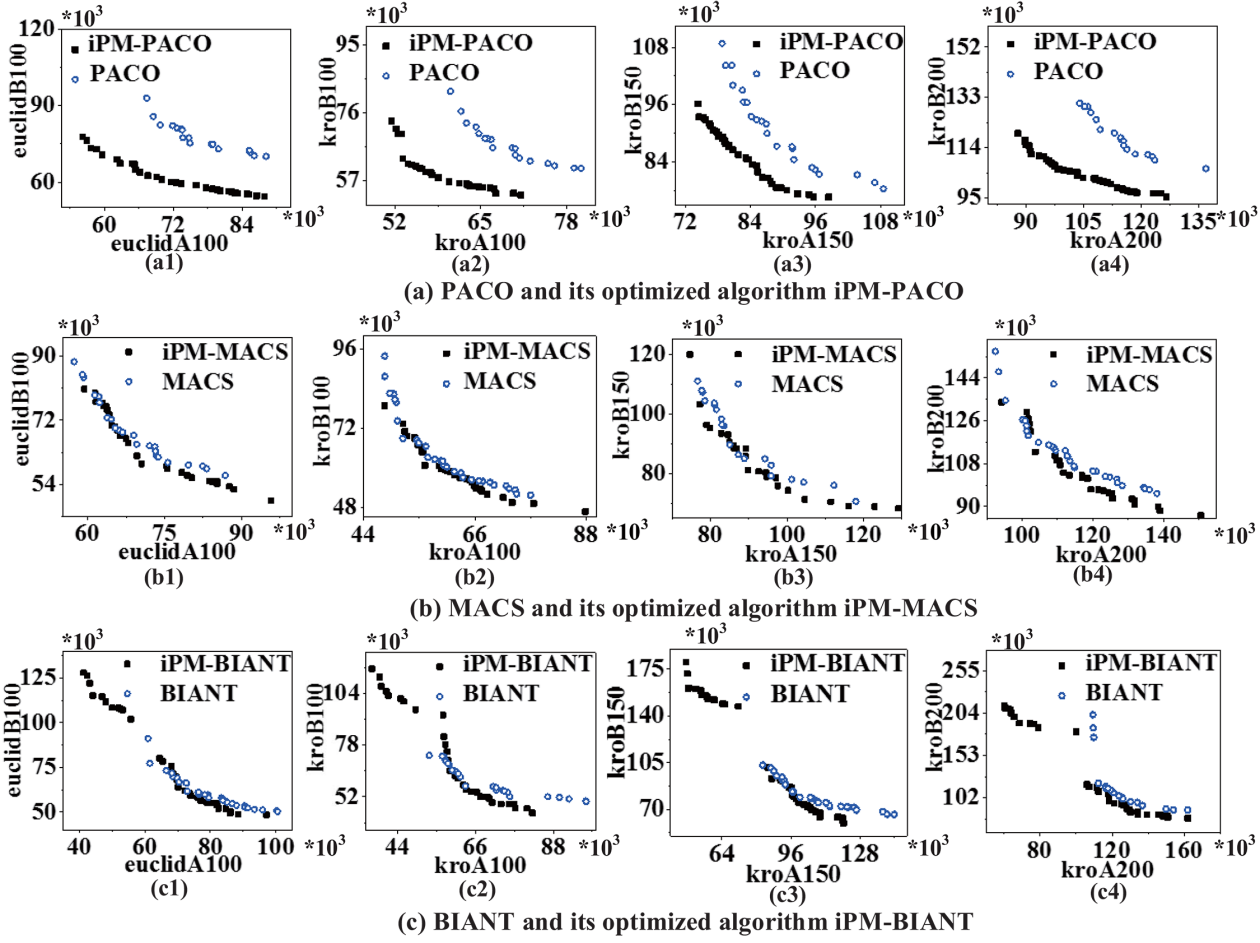
PFs returned by MOACOs and iPM-MOACOs in four bi-objective symmetric TSP instances. (a) PACO and iPM-PACO, (b) MACS and iPM-MACS, (c) BIANT and iPM-BIANT. From left to right, the instances are euclidAB100, kroAB100, kroAB150 and kroAB200. Results show that most of solutions generated by iPM-MOACOs in four instances can dominate the solutions generated by MOACOs, which mean that iPM-MOACOs can obtain better PF than that of MOACOs. Specially, the distribution of solutions generated by iPM-BIANT is better than that of BIANT, as shown in (c).

Fig 5 plots the graphical representation of PFs returned by MOACOs and the iPM-MOACOs in four instances, where each coordinate represents an objective, and each point corresponds to a feasible solution for the instance. All PFs generated by each algorithm will be fused into a single PF by removing the dominated solutions. This result shows that the optimized strategy for updating pheromone matrix can improve the quality and distribution of solutions significantly, especially for PACO.

**Fig 6:**
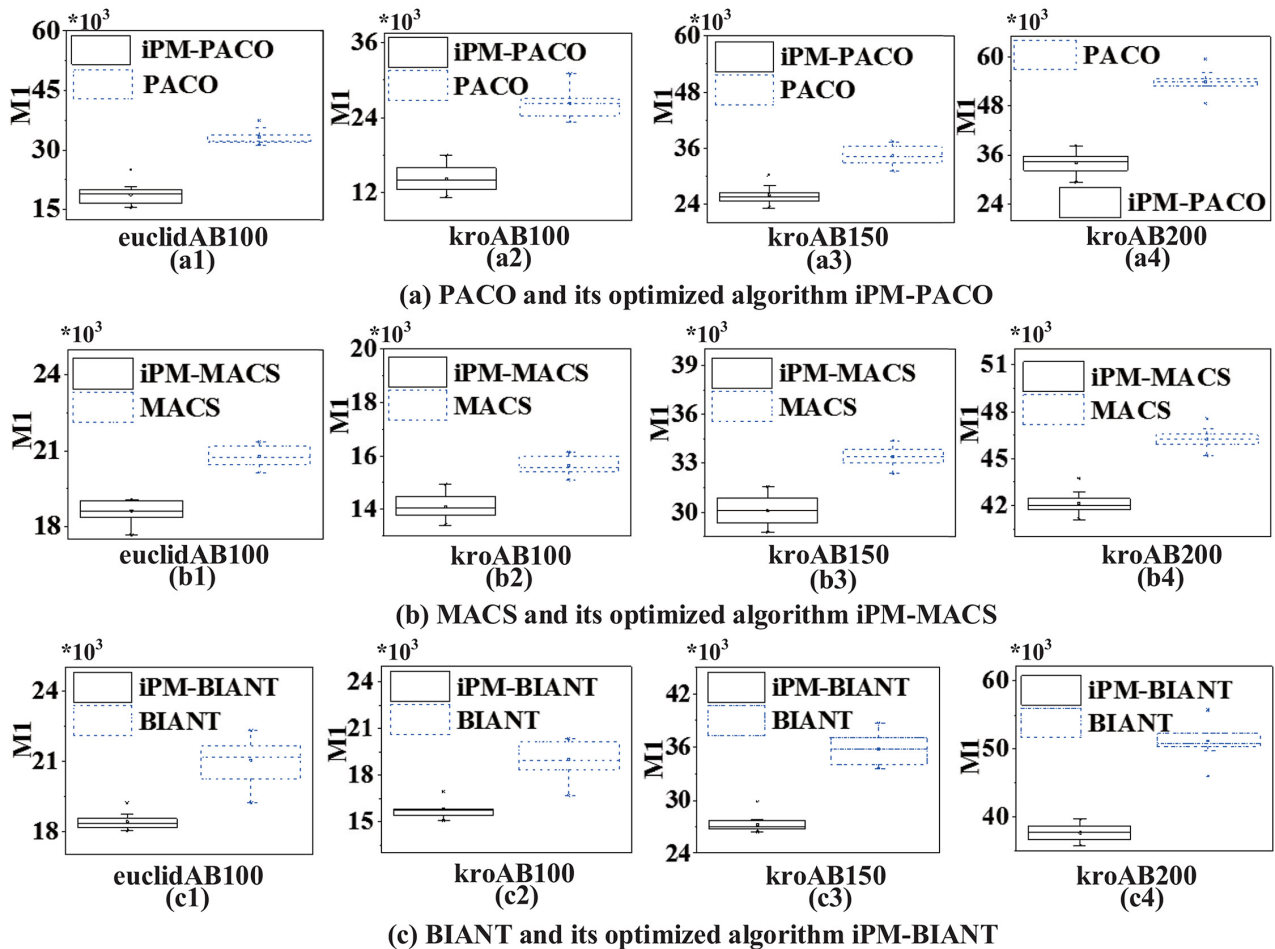
*M*1 metric comparison between MOACOs and iPM-MOACOs in four instances. From left to right, the instances are euclidAB100, kroAB100, kroAB150 and kroAB200. Results show that each corresponding *M*1 values of optimized MOACOs are much lower than those of MOACOs in four instances, which means that solutions generated by the optimized MOACOs are much closer to the pseudo-optimal PFs.

**Fig 7:**
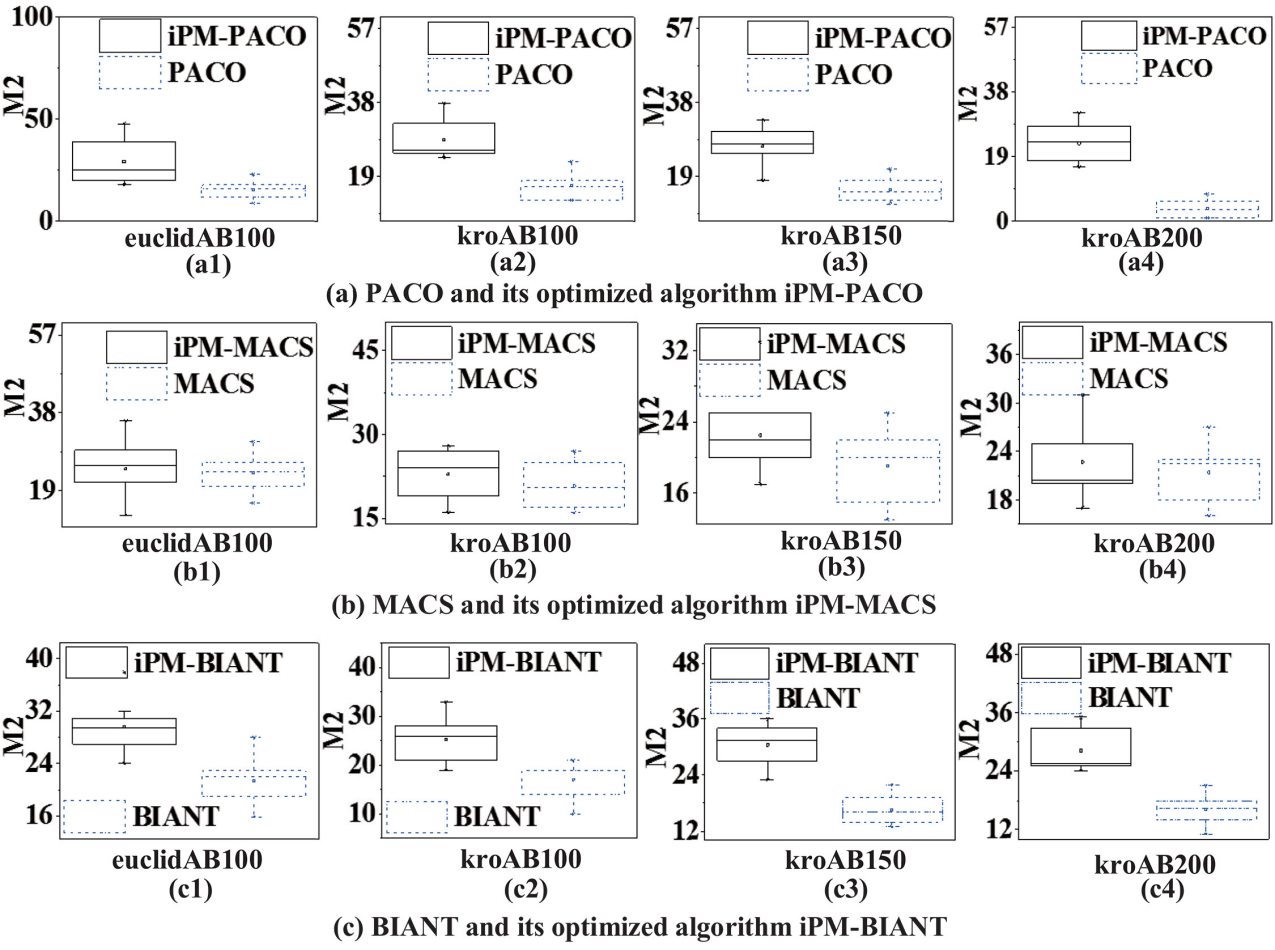
*M*2 metric comparison between MOACOs and iPM-MOACOs in four instances. From left to right, the instances are euclidAB100, kroAB100, kroAB150 and kroAB200. Results show that the each corresponding *M*2 values of iPM-MOACOs are more reasonable than those of the corresponding original algorithms.

**Fig 8:**
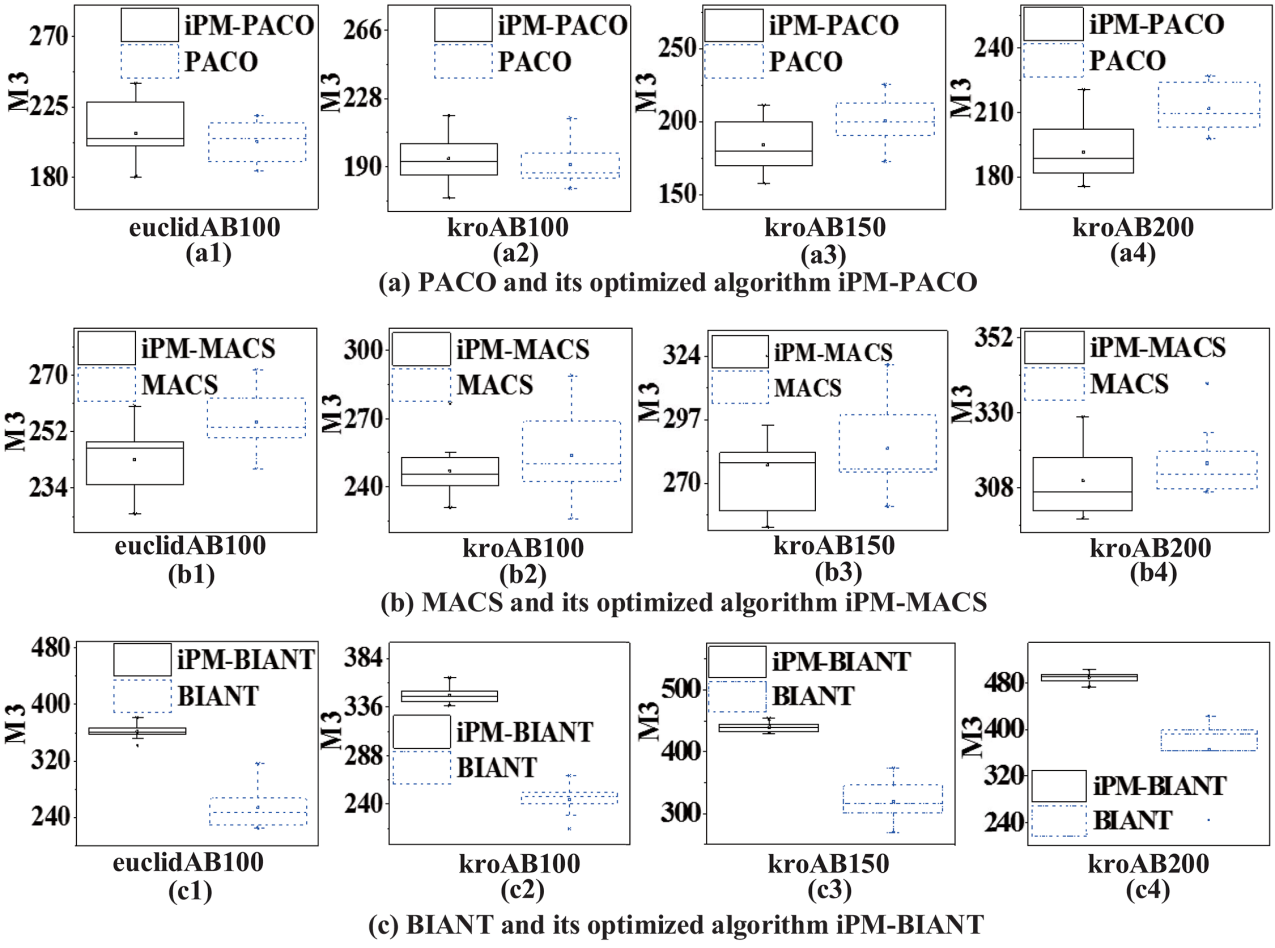
*M*3 metric comparison between MOACOs and iPM-MOACOs in four instances. From left to right, the instances are euclidAB100, kroAB100, kroAB150 and kroAB200. According to these results, we know that most of *M*3 metrics of iPM-MOACOs are better than those of original MOACOs, especially for BIANT.

In order to further compare the performances between MOACOs and iPM-MOACOs quantitatively, box-plots in Figs 6, 7 and 8 are used to estimate the values of *M*1, *M*2 and *M*3 metrics. In each box, the highest and lowest lines represent the maximum value and minimum value with 10 runnings, respectively. The upper and lower ends of a box are the upper and lower quartiles, respectively. The line within a box means the median of solutions.

Fig 6 shows that PFs generated by the optimized algorithms (i.e., iPM-MOACOs) are much closer to the pseudo-optimal PFs. Fig 7 evaluates the distribution of solutions in PFs returned by the original algorithms (i.e. MOACOs) and the optimized (i.e., iPM-MOACOs) according to *M*2 indicator. Results show that iPM-MOACOs can obtain a better distribution of solutions. Furthermore, we estimate the extent of solutions by comparing *M*3 metic. As plotted in Fig 8, the extent of solutions of PM-MOACOs are better than the original MOACOs.

Table 3 demonstrates the values of *C* metric in four bi-objective symmetric TSP instances. Each value represents the fraction of algorithm *A*2 covered by algorithm *A*1 (*C*(*A*1, *A*2)). For example, for instance euclidAB100, *C*(iPM-PACO, PACO) = 1.0000, which means that the PF generated by iPM-PACO dominates the PF generated by PACO with the probability of 100%. According to Table 3, we can draw the conclusion that all non-dominated solutions of PACO are dominated by those of iPM-PACO. Meanwhile, most of non-dominated solutions of MACS and BIANT are dominated by those of correspondent optimized algorithms. The results are corresponding with the graphic representation of PFs in Fig 5, i.e., the iPM-MOACOs perform better than MOACOs.

**Table 3:** *C* metric comparison results between MOACOs and PM-MOACOs.

Instances	*C*(PM-PACO, PACO)	*C*(PM-MACS, MACS)	*C*(PM-BIANT, BIANT)
euclidAB100	1.0000	0.6522	0.8125
kroAB100	1.0000	0.9000	0.7083
kroAB150	1.0000	0.8000	0.8065
kroAB200	1.0000	0.7667	1.0000

#### • Comparisons among MOACOs, PM-MOACOs and iPM-MOACOs

In order to validate the performance of our updating strategy, a series of experiments are implemented among PACO, PM-PACO (in which the optimization strategy is implemented in each iteration as shown in [19]) and iPM-PACO.

**Fig 9:**
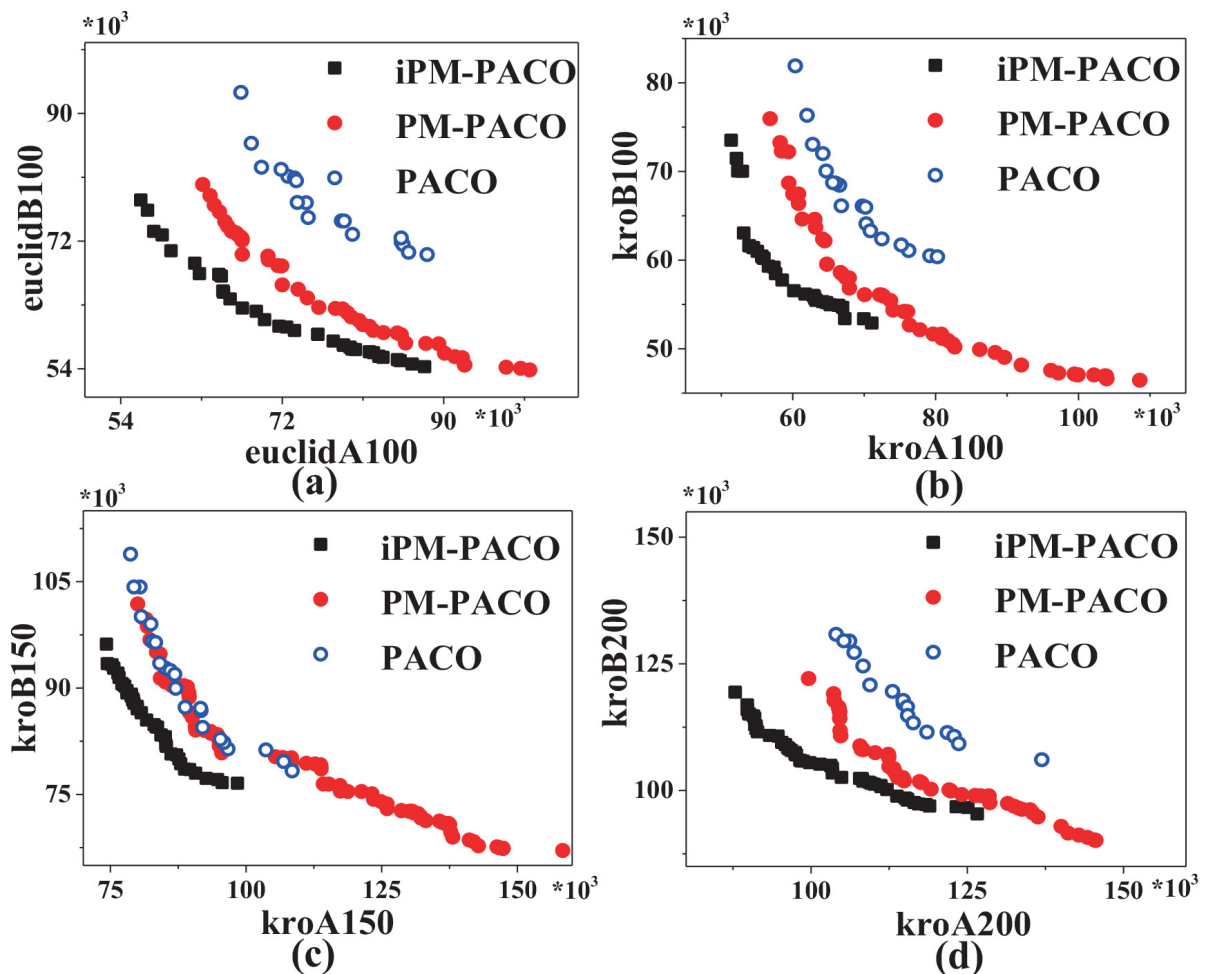
PFs returned by PACO, PM-PACO and iPM-PACO in four benchmark instances. From left to right, the instances are euclidAB100, kroAB100, kroAB150 and kroAB200. The results show that most of solutions generated by iPM-PACO can dominate the solutions generated by PM-PACO and PACO. Since the distributions of solutions generated by PM-PACO are better than that of iPM-PACO, the solutions of PM-PACO are not dominated by solutions of iPM-PACO in the bottom-right regions of the PFs.

**Fig 10:**
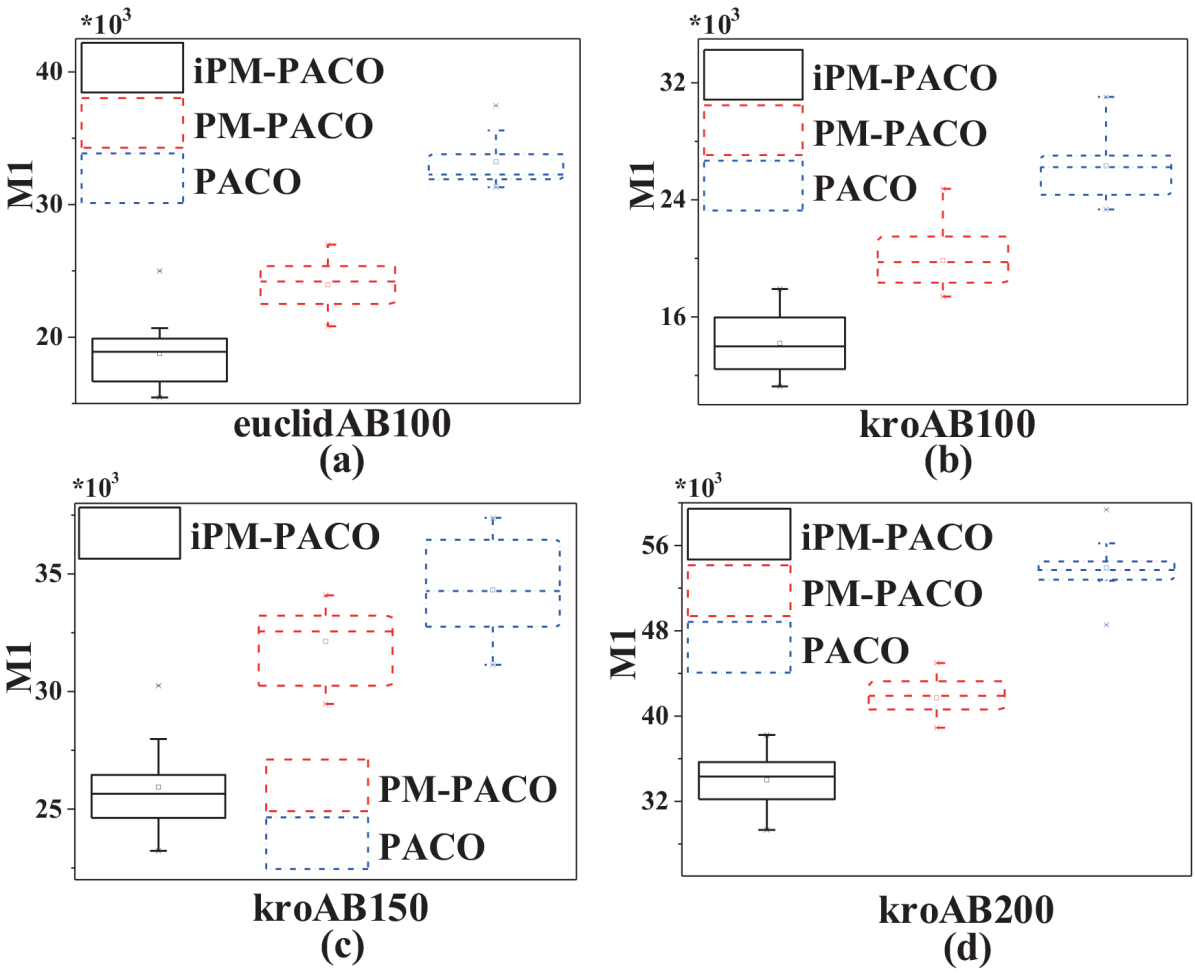
*M*1 metric comparisons among PACO, PM-PACO and iPM-PACO in four instances. From left to right, the instances are euclidAB100, kroAB100, kroAB150 and kroAB200. According to these results, each corresponding *M*1 values of iPM-PACO is the lowest, meanwhile, each corresponding *M*1 values of PACO is the highest. Results show that solutions generated by iPM-PACO are the closest to the pseudo-optimal PFs.

**Fig 11:**
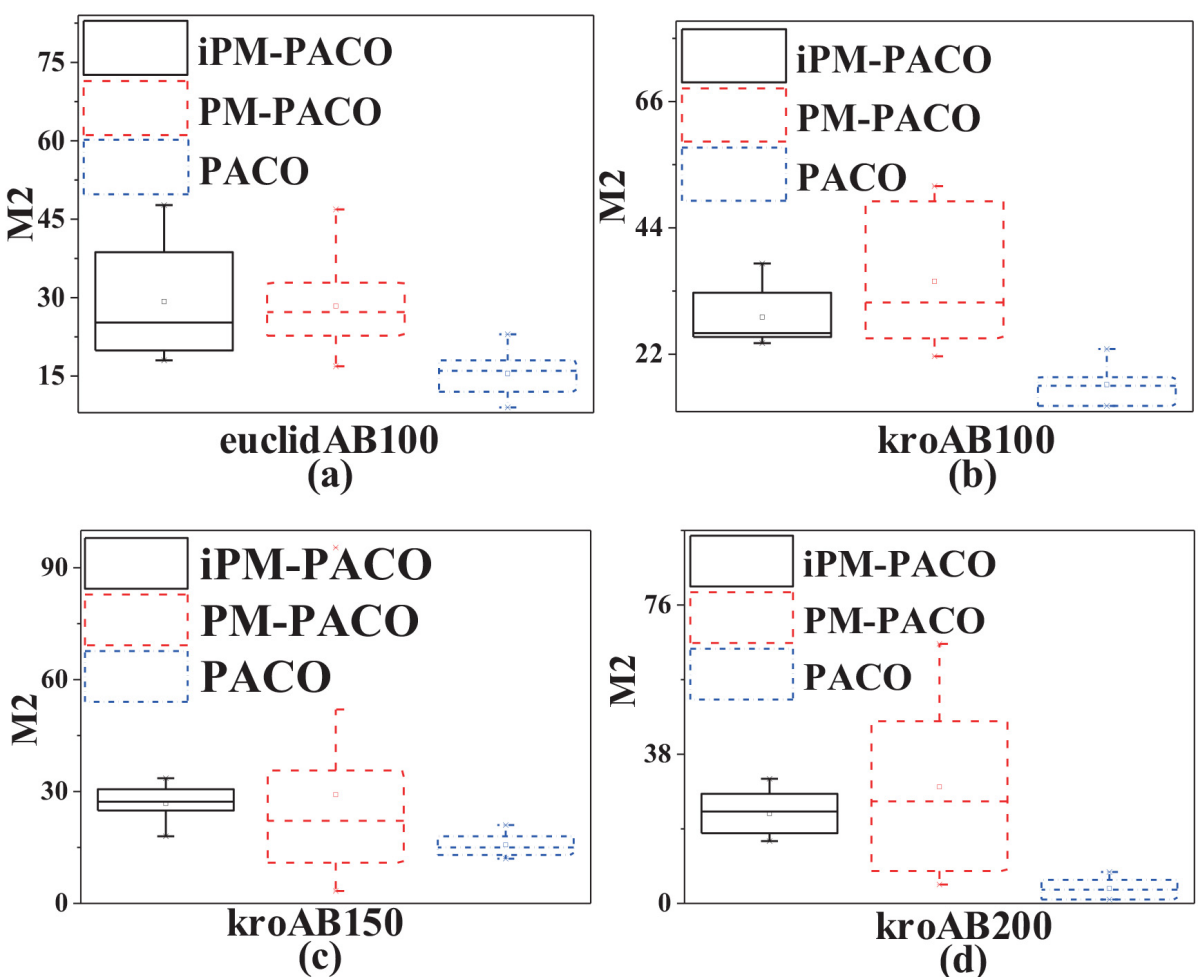
*M*2 metric comparison between PACO, PM-PACO and iPM-PACO in four instances. From left to right, the instances are euclidAB100, kroAB100, kroAB150 and kroAB200. Results show that each corresponding *M*2 values of PACO is the lowest. Meanwhile, box-plots of PM-PACO are longer than those of iPM-PACO. Meanwhile, the distribution of PACO is the narrowest, and the distribution of iPM-PACO is more stable than PM-PACO.

**Fig 12:**
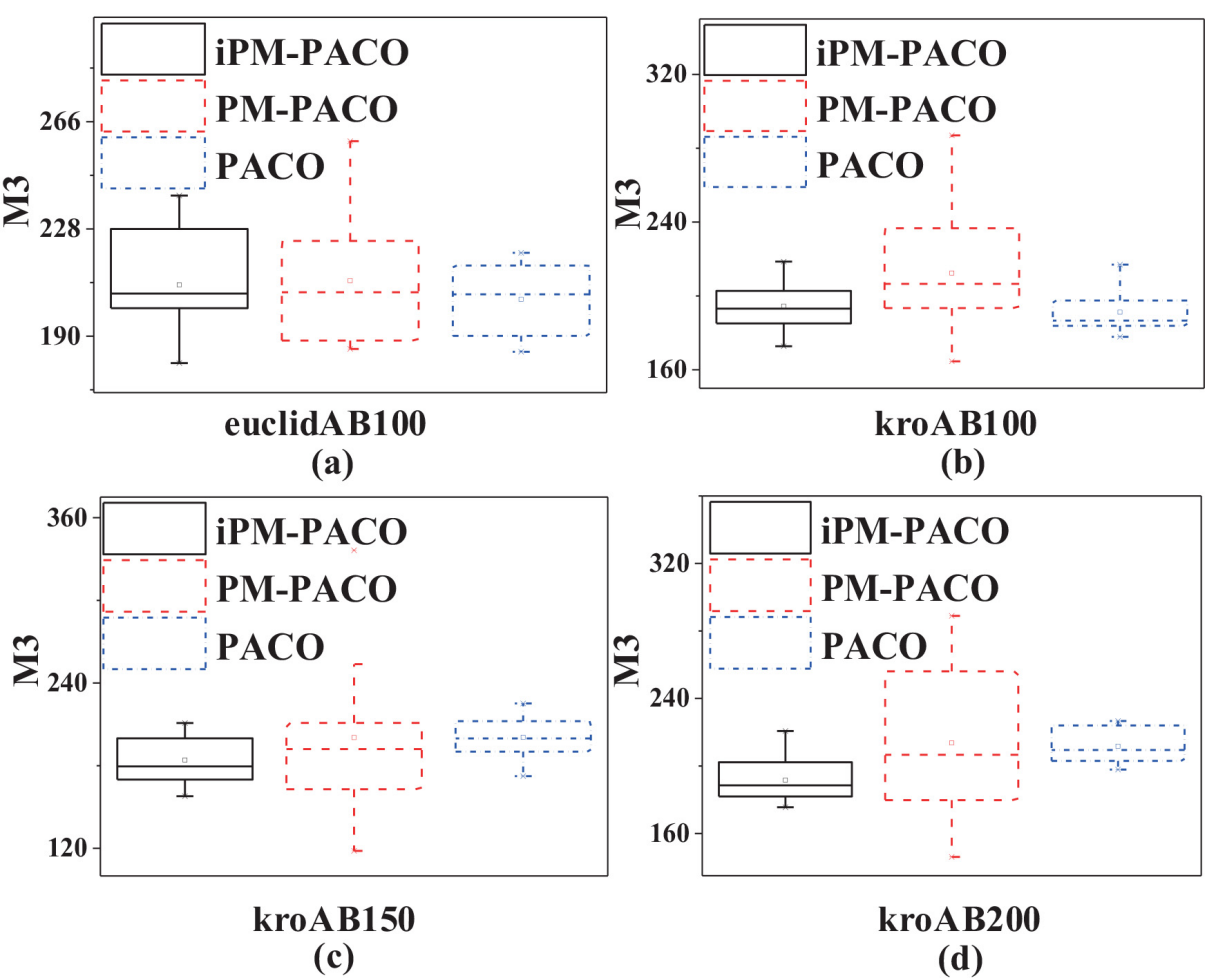
*M*3 metric comparison between PACO, PM-PACO and iPM-PACO in four instances. From left to right, the instances are euclidAB100, kroAB100, kroAB150 and kroAB200. This figure shows that *M*3 values of iPM-PACO and PACO are close, and those of PM-PACO are slightly higher. Results show that the extent solutions of three algorithms are approximate.

Fig 9 plots that solutions obtained by iPM-PACO are the most accurate among three algorithms. Meanwhile, solutions calculated by PM-PACO have the widest distribution among these algorithms, and solutions obtained by PM-PACO are more accurate than those obtained by PACO. As shown in Fig 10, solutions generated by iPM-PACO are the closest to the pseudo-optimal PFs. Furthermore, PFs obtained by PM-PACO are much closer to the pseudo-optimal PFs than those obtained by PACO. And, the distribution of solutions in the PF returned by iPM-PACO is the best as shown in Fig 11. Fig 12 illustrates that the extents of solutions calculated by three algorithms are similar, and PM-PACO is slightly better in the extent of solutions. According to these measurements, we can summarize that iPM-PACO is the best algorithm in accuracy and distribution of solutions among the three algorithms, and three algorithms perform similarly in the spread of solutions.

The value of *C* metric in four instances can be found in Table 4. we can draw the conclusion that all non-dominated solutions generated by PACO are dominated by those generated by iPM-PACO, while most of non-dominated solutions generated by PM-PACO are dominated by those generated by iPM-PACO. Results are corresponding with the graphic representation of PFs in Fig 9, i.e., iPM-PACO performs the best efficency among three algorithms.

**Table 4:** *C* metric comparison results among PACO, PM-PACO and iPM-PACO.

Instances	*C*(iPM-PACO, PM-PACO)	*C*(iPM-PACO, PACO)	*C*(PM-PACO, PACO)
euclidAB100	0.9400	1.0000	1.0000
kroAB100	0.4615	1.0000	1.0000
kroAB150	0.5417	1.0000	0.5909
kroAB200	0.8730	1.0000	1.0000

According to these experimental results, the extent solutions calculated by iPM-PACO and PM-PACO are approximate. However, iPM-MOACOs are better than PM-MOACOs in accuracy and distribution of solutions with the effect of prior knowledge decreasing. Because the unchanged priori knowledge of PMM implements with the two pheromone matrices in each iteration, PM-PACO may get the local optimal solutions and fall into the narrow search fields.

### (3) Discussion

There are different ways to measure the quality of the solutions of bTSP [18]. Recently, a very popular measure is the hypervolume indicator [25, 26], which incorporates both the optimality of a solution set, as well as its spread in the objective space [27, 28]. It is of exceptional interest as it possesses the highly desirable feature of strict Pareto compliance [29], which means that there are two Pareto sets, *A* and *B*, the Pareto sets *A* dominates the Pareto sets *B* only when the hypervolume indicator values of *A* is higher than that of *B* [30]. Eq (28) is the definition of hypervolume indicator, where *HV* is the hypervolume indicator, in which R and *X* are the objective space and the current Pareto set, respectively. And *r* is the point which belongs to *X*, *z* is the given reference point, and *μ* is the Lebesgue measure on R [31].

$$\begin{array}{c} HV(X;z)=\mu (\{x\in R:\exists r\in X\;s.t.\;r≺x≺z\}) \end{array}$$ (28)

Hypervolume indicator is more applicable than Pareto compliance, because it can measure PFs without Pareto-optimal front that is rarely known [21]. In order to make sure that the optimized strategy is better than the original in hypervolume indicator, we implement some experiments to compare the *HVs* of solutions above. Meanwhile, we construct a bTSP instance, called euclidAC100, which is constructed using euclidA100 and euclidC100 according to [12]. Specially the benchmarks, labeled as euclidA100 and euclidC100, are also available on Jaskiewicz’s web page (https://eden.dei.uc.pt/~paquete/tsp/). There is no Pareto-optimal front for euclidAC100. Table 5 is the comparison results of *HV* between MOACOs and PM-MOACOs, and these *HV*s show that most of results of optimized strategy are higher than the original except results of PM-MACS in kroAB100 and PM-BIANT in euclidAC100, which is a little lower than results of original algorithms. We can draw the conclusion that the optimized strategy performs better than the original in hypervolume indicator in most cases.

**Table 5:** *HV* comparison results between MOACOs and iPM-MOACOs.

Algorithms	kroAB100	kroAB150	kroAB200	euclidAB100	euclidAC100
PACO	1.39E + 10	1.68E + 10	1.53E + 10	1.80E + 10	0.61398e + 010
PM-PACO	1.88E + 10	2.80E + 10	3.51E + 10	2.55E + 10	1.21896e + 010
BIANT	1.01E + 10	1.57E + 10	1.61E + 10	1.46E + 10	1.59319e + 010
PM-BIANT	1.88E + 10	3.12E + 10	3.00E + 10	1.97E + 10	1.20890e + 010
MACS	1.79E + 10	1.66E + 10	1.86E + 10	1.30E + 10	1.64082e + 010
PM-MACS	1.65E + 10	2.34E + 10	2.17E + 10	1.62E + 10	2.08092e + 010

**Fig 13:**
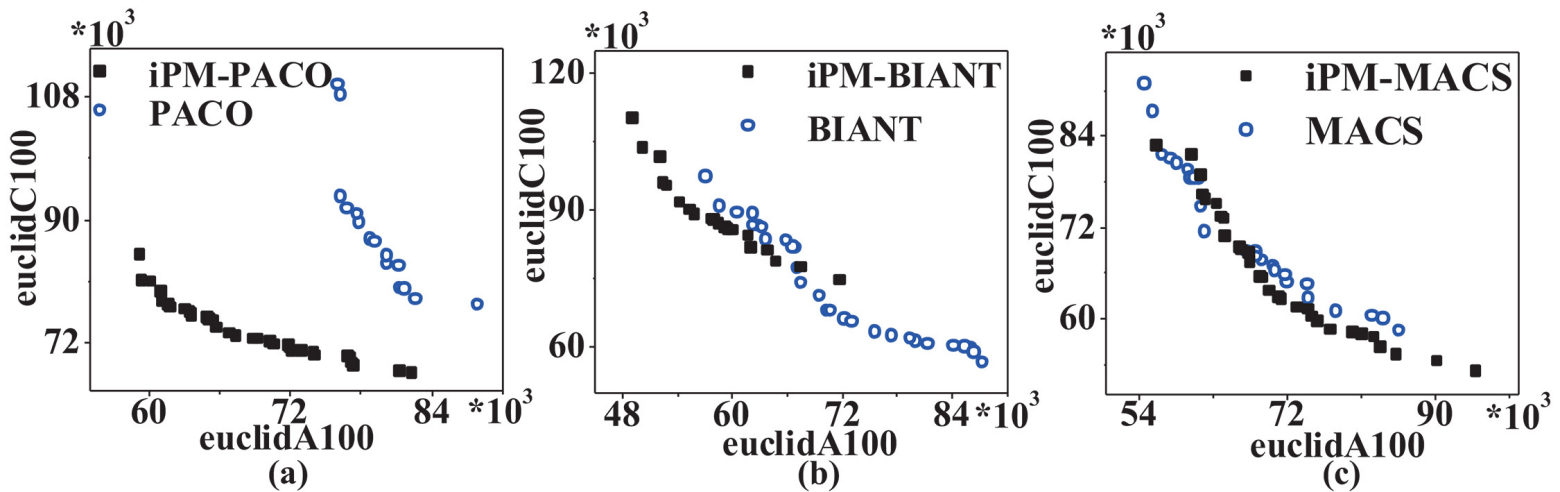
PFs returned by MOACOs and iPM-MOACOs in euclidAC100 instances. (a) shows that the PF generated by PACO always converges to the top-left regions, while the PF calculated by the optimized PACO converges to bottom-left regions. Meanwhile, (b) performs that most of intersecting points of PF obtained by the optimized BIANT aggregate in the bottom-left regions when compared with those obtained by the original BIANT. (c) displays that the PF returned by the optimized MACS is closed to the PF calculated by the original. The comparisons show that solutions obtained by the optimized algorithms are more reasonable.

Fig 13 is PFs calculated in euclidAC100, we can see that the PF returned by iPM-PACO dominates the PF returned by PACO. More specifically, points of PF calculated by iPM-BIANT dominate most of points of PF calculated by BIANT in the area where they intersect, and points of PF generated by iPM-MACS are close to ones of PF generated by MACS. It matches results obtained by the corresponding *HVs*.

## Conclusions

In this paper, we propose a new updating strategy based on PMM, which takes advantage of the prior knowledge of PMM to optimize the initialization of MOACOs. Due to the effect of positive feedback information of PMM, iPM-MOACOs can promote the exploitation of optimal solution. Meanwhile, compared with PM-MOACOs, iPM-MOACOs have a wider search scope, because the effects of optimization will reduce with the increment of iteration based on the evaporation of pheromone. Some experiments in bi-objective symmetric TSP instances are conducted and five typical measurements are utilized for comparison. The experimental results show that PFs obtained by iPM-MOACOs are closer to the pseudo-optimal PFs, and have a better distribution and wider extent comparing with PFs obtained by MOACOs. Furthermore, in order to validate the superiority of iPM-MOACOs in bTSPs without Pareto-optimal front, the comparison results measured by hypervolume indicator are discussed. Results show that most of *HV*s obtained by the new updating strategy are higher than those obtained by the original. According to these experimental results, we can conclude that the quality of solutions generated by iPM-MOACOs are better than that of the original MOACOs.
